# Protein–DNA Interactions Regulate Human Papillomavirus DNA Replication, Transcription, and Oncogenesis

**DOI:** 10.3390/ijms24108493

**Published:** 2023-05-09

**Authors:** Roxanne Evande, Anshul Rana, Esther E. Biswas-Fiss, Subhasis B. Biswas

**Affiliations:** Department of Medical and Molecular Sciences, University of Delaware, Newark, DE 19716, USA

**Keywords:** HPV, cervical cancer, oropharyngeal cancer, E2 protein

## Abstract

Human papillomavirus (HPV) is a group of alpha papillomaviruses that cause various illnesses, including cancer. There are more than 160 types of HPV, with many being “high-risk” types that have been clinically linked to cervical and other types of cancer. “Low-risk” types of HPV cause less severe conditions, such as genital warts. Over the past few decades, numerous studies have shed light on how HPV induces carcinogenesis. The HPV genome is a circular double-stranded DNA molecule that is approximately 8 kilobases in size. Replication of this genome is strictly regulated and requires two virus-encoded proteins, E1 and E2. E1 is a DNA helicase that is necessary for replisome assembly and replication of the HPV genome. On the other hand, E2 is responsible for initiating DNA replication and regulating the transcription of HPV-encoded genes, most importantly the E6 and E7 oncogenes. This article explores the genetic characteristics of high-risk HPV types, the roles of HPV-encoded proteins in HPV DNA replication, the regulation of transcription of E6 and E7 oncogenes, and the development of oncogenesis.

## 1. Introduction

Human papillomavirus (HPV) is one of the most common sexually transmitted infections, affecting millions worldwide annually. Human papillomaviruses belong to the papillomaviridae family of small, non-enveloped, double-stranded DNA viruses [1], which includes a large number of species-specific genotypes that predominantly infect the cutaneous and mucosal epithelium in various organisms [2,3]. The host’s immune system quickly clears most HPV infections within a couple of years. However, some HPV types cause persistent infections and become the causative agents of various genital and oropharyngeal cancers [4]. Currently, ~160 HPV strains, commonly called “types”, have been identified. These types are often classified into low- and high-risk types based on their propensity to induce cancer. Three vaccines against HPV are available, but no more than nine HPV types are covered by the latest vaccine: 6, 11, 16, 18, 31, 33, 45, 52, and 58. Of note, there are currently no treatments available for HPV infection. HPV accounts for approximately half a million deaths per year around the world. It is a silent viral epidemic.

## 2. The HPV Genome

**Figure 1:**
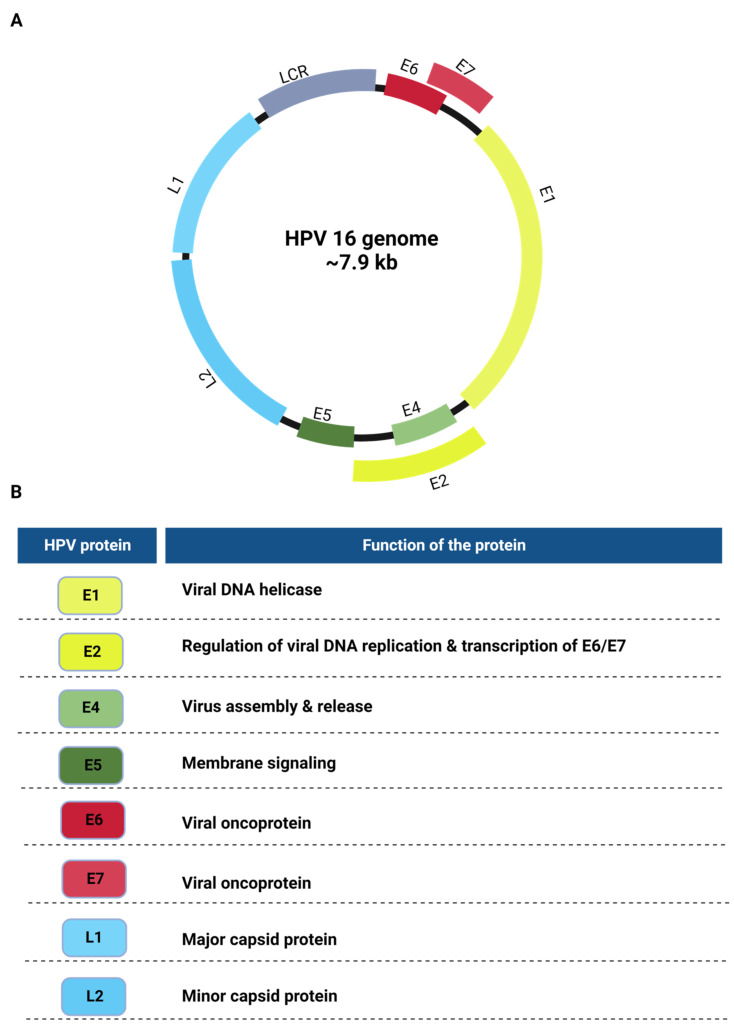
(**A**). **A genetic map of the HPV genome**. The HPV genome is ~8 kb in length and encodes eight major genes separated by their expression order during the life cycle. (**B**). **List depicting the major functions of each of the HPV proteins**.

The HPVs have double-stranded circular DNA genomes (~8 kb) containing eight open reading frames (ORFs) that encode viral proteins [5]. There is a noncoding region in the HPV genome called the long control region (LCR), which contains the origin of DNA replication and transcriptional regulatory elements (Figure 1A) [5]. The genome is divided into three main sections: the early region, containing the early genes; the late region, containing the late genes; and the long control region (LCR) [6]. The first six genes encode the early viral proteins and are expressed at the beginning of the viral life cycle. Of these viral proteins, E1 and E2 are the major replication proteins, E4 and E5 aid in genome amplification, and the E6 and E7 proteins are the oncoproteins (Figure 1). The late genes, L1 and L2, encode the L1 major and L2 minor capsid proteins. The L1 and L2 proteins together form the capsid. They are expressed in the later stages of the viral life cycle. The LCR is the only noncoding region of the genome. It contains the early viral promoters, enhancers, and the origin of viral DNA replication [7,8].

The E2 protein can be considered the most pivotal protein in the HPV genome due to its role in the viral life cycle and oncogenesis. The HPV viral life cycle is tightly controlled by the E2 protein, which is also involved in transcriptional regulation, the expression of the E6 and E7 oncogenes, partitioning, and the maintenance of the viral genome, in addition to its role in the initiation of DNA replication [4]. 

## 3. Evolution and Diversity of HPV Types

Papillomaviruses are ancient DNA viruses spanning ~400 million years and have been identified in humans, nonhuman primates, bovines, and other animals such as dolphins [9]. HPVs have significantly diverged and evolved genetically and phenotypically over time. In the early 1900s, papillomaviruses were discovered in humans (HPV) and subsequently associated with cervical cancer by zur Hausen in 1975 [10]. 

There are five major known HPV genera: α (alpha), β (beta), γ (gamma), μ (mu), and υ (nu) [11]. Carcinogenic HPV types belong to the alpha or beta genus and infect the mucosal epithelium [12,13]. Recent studies have also shown the association of gamma HPV types with various oropharyngeal cancers [14,15].

**Figure 2:**
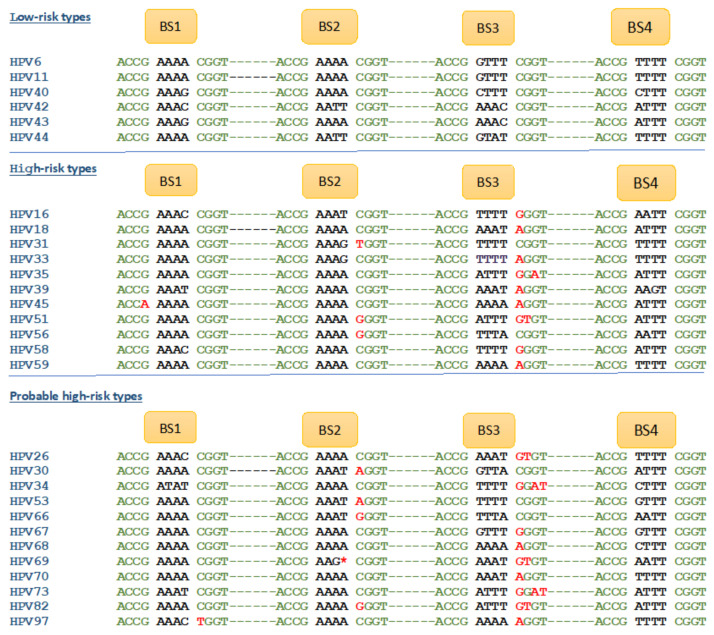
**Sequence variations of E2 binding sites correlate with cancer risks**. E2 binding sites and detection of SNVs in the consensus sequence associated with clinically well-characterized HPV types. Variant nucleotides in the consensus sequence are indicated in red.

The IARC working groups have classified many of the HPV types into three categories, low-risk, high-risk, and probable high-risk (Figure 2), leaving many HPV types unclassified. Low-risk HPV types commonly refer to the HPV types that cause anogenital warts and benign lesions. These types are generally non-lethal. High-risk HPV types commonly refer to the HPV types that have greater oncogenic potential and cause carcinomas [12,16,17]. Overall, 90% of cervical cancers are due to high-risk HPV infection. HPV16 and 18 are the most common high-risk HPV types and account for 70% of all cervical cancer cases [16].

Yilmaz et al. discovered nucleotide variations in the E2 binding site consensus sequences (ACCGNNNNCGGT) of at least one of the three binding sites in all established high-risk HPV types (Figure 2) [18]. All HPV types classified as low-risk on the Papillomavirus database (PaVe) have intact consensus sequences. However, all high-risk or probable high-risk HPV types had at least one nucleotide variation in their consensus sequence (Figure 2). Using in vitro DNA binding assays with oligonucleotides containing normal and variant consensus sequences, this study established the attenuation of E2–DNA complex formation upon consensus sequence variations. Thus, the nucleotide variations affected HPV E2 binding to its binding sites, affecting the biological functions of E2 in HPV. This model can potentially be used to predict the oncogenic potential of all HPV types based on the DNA sequence of HPV.

As the Yilmaz classification of HPV types is based on the DNA sequence, any HPV type can be classified based on the consensus sequence mutation(s), without requiring clinical data on the potential to cause cancer, as shown in Figure 2 [18]. For example, HPV97, 102, and 114 are currently unclassified in terms of oncogenicity, but these HPV types contain variant E2 binding sites, making them high-risk.

## 4. Regulation of DNA Replication, Transcription, and Oncogenesis by E2 Protein

**Figure 3:**
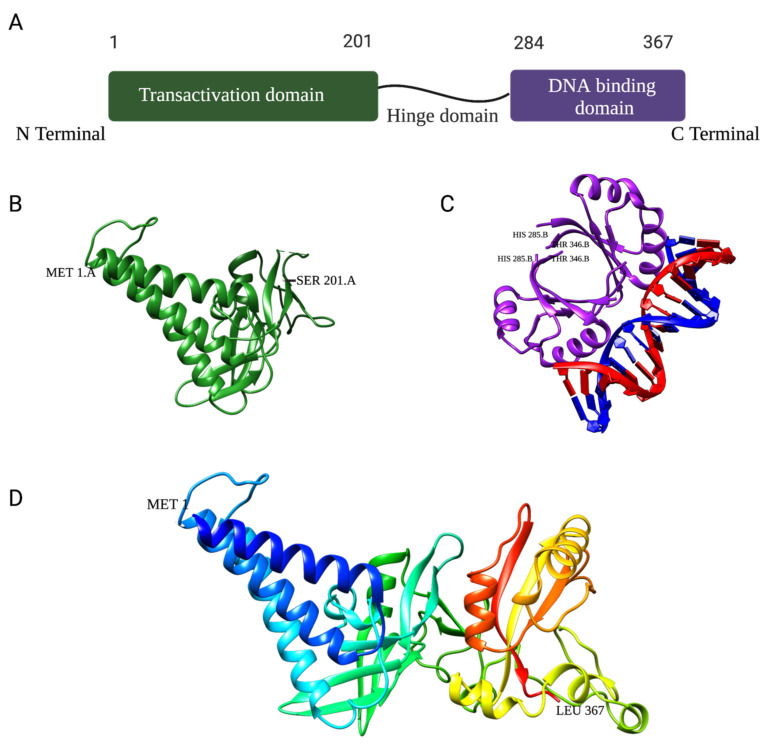
(**A**). **Schematic structure of the HPV16 E2 protein** and its respective domains: transactivation, hinge, and DNA binding. (**B**). Ribbon representation of the HPV16 E2 transactivation domain crystal structure (PDB: 1DTO). (**C**). Ribbon representation of the crystal structure of HPV18 E2 DNA-binding domain as a dimer bound to E2 binding site 4 with a helix from each monomer interfaced with ACCG/CGGT motif (PDB: 1JJ4). (**D**). Homology model of the full-length HPV11 E2 protein monomer using Robetta Structure Prediction software from the University of Washington (25 April 2023).

The HPV E2 protein is a DNA-binding protein with two conserved functional domains. The N-terminal has the transactivation domain, and the C-terminal has the DNA-binding domain. The two domains are connected by a flexible linker known as the “hinge region” (Figure 3A) [18,19]. 

The transactivation domain is the largest, with approximately 200 amino acids, and is required for its replication, transactivation, and segregation functions (Figure 3B). The hinge region is serine–arginine-rich and serves as a linker between the two domains. Finally, the DNA-binding domain interacts with sequence-specific binding sites within the long control region (LCR) and binds as a dimer (Figure 3C) [20,21]. To date, the crystal structure of the full-length HPV E2 protein has yet to be determined. 

Various papillomaviruses have shorter, truncated E2 proteins, known as isoforms. In the bovine papillomavirus, truncated E2 isoforms were observed and determined to be repressors of transcription and replication [22]. In HPV, several types, such as HPV16, have isoforms predicted to have been generated through alternative splicing: E1^E2 and E8^E2. These E2 isoforms have a domain whose parts are either missing or misplaced. These alterations of E2 may result in similar protein functions but at a reduced capacity. For example, the E2/E8^E2 dimer has a truncated transactivation domain. While this might indicate regular E2 DNA binding, the recruitment of E1 might not occur [6]. Other studies have shown that, as in BPV, HPV16 E8^E2 can repress early transcription and replication [23]. All isoforms observed to date still maintain their replicational or transcriptional functions but not the partitioning of the viral genome. This could indicate that the partitioning function always requires a full-length E2 protein.

### 4.1. E2 Binding Sites in the HPV Genomes

#### 4.1.1. E2 Binding to Four Binding Sites (BS1–4) of the LCR

**Figure 4:**
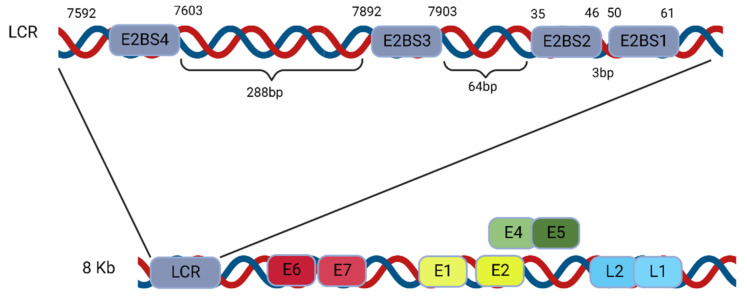
Illustration showing the location and structure of the viral DNA replication origin in the HPV genome. The enlargement of the LCR indicates the locations of the four E2 binding sites (BS1–4) (not drawn to scale).

The HPV E2 protein recognizes a consensus 12 bp palindromic sequence, and a sequence of four such binding sites is located in the long control region (LCR), sometimes referred to as the upstream regulatory region (URR). The LCR is the noncoding region of the HPV genome, and within it lie various binding sites, the origin of replication, and a few other cis elements. These four E2 binding sites (BS1–4) are highly conserved across papillomaviruses and are spatially arranged and characterized by the 12 bp palindromic sequence ACCG (N_4_)CGGT, where N denotes any nucleotide (Figure 4) [24]. 

The DNA-binding domain located at the C-terminus of the E2 protein (Figure 3B) recognizes and binds to these four palindromic binding site sequences but with varying affinities (Table 1). Specifically, the low-risk HPV11 E2 protein has the strongest binding affinity for the BS4 site in HPV11, with a dissociation constant (KD) of 3.9 ± 0.5 nM; somewhat weaker binding affinities to binding sites 1 and 2, with KD around 5.8 ± 0.7 nM and 5.3 ± 0.4 nM, respectively; and the lowest binding affinity is observed with binding site 3, with a KD of 8.9 ± 0.9 nM (Table 1) [18,25,26,27].

#### 4.1.2. E2 Binding to Its Binding Sites at the LCR Modulates DNA Replication Initiation

The interaction between the HPV E2 protein and these binding sites is essential for E2 to regulate several functions in the genome. The E2 protein initiates DNA replication by binding to its sites, represses transcription of the oncogenes, and consequently halts oncogenesis. It’s been demonstrated that a single nucleotide variation in the consensus sequences can play a role in the fate of HPV-infected cells and help determine whether they will become a high or low risk for cancer. This interaction between the E2 protein and its binding sites plays an important role in the oncogenicity of high-risk HPVs. The E2 protein binds to all four binding sites but with different affinities [18]. As demonstrated in a binding study, a single nucleotide variation (SNV) in any of these binding sites can lead to reduced binding affinity to no binding at all. Using in vitro DNA binding assays with oligonucleotides containing normal and variant consensus sequences, this study established attenuation of E2-DNA complex formation upon consensus sequence variations. Thus, the nucleotide variations affected HPV E2 binding to its binding sites affecting the biological functions of E2 in HPV.

**Figure 5:**
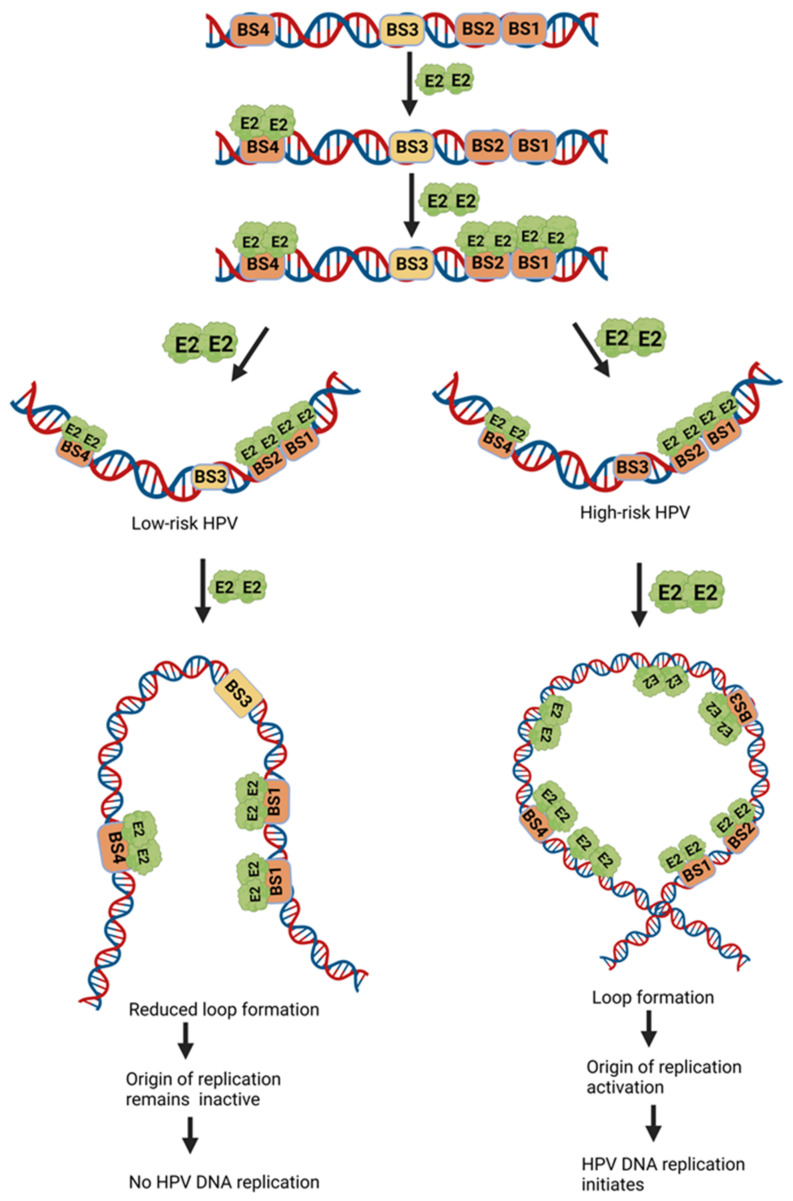
**Putative models for HPV E2 DNA binding and activation of the low-risk and high-risk replication origins**. The E2 protein in low-risk HPV binds to its specific sequence and creates a loop formation to initiate DNA replication. The E2 protein in high-risk HPV binds the sequence with weaker affinity, thus decreasing the loop formation and inactive origin, which leads to no replication of viral DNA.

Based on these binding studies, Yilmaz et al. have proposed a potential model for the E2 protein binding and activation at the low-risk and high-risk origins [18]. According to the model (Figure 5), an SNV in BS2 and/or BS3, observed in high-risk HPV types exclusively, can lead to inefficient binding of the E2 protein (Table 1), thereby causing inefficient DNA replication. The low-risk HPVs, with no SNV, replicate better than the high-risk virus [18]. 

### 4.2. HPV Life Cycle and Regulation of DNA Replication

The replication and life cycle of HPV are initiated and controlled by epithelial cell differentiation [28]. HPV enters the stratified epithelium through a cut, abrasion, or wound, where it integrates into cells at the basal layer. 

Exposure of the basement membrane allows the L1 capsid protein of virions to bind to the heparin sulphate proteoglycans (HSPGs) present on the surface of the basal keratinocytes for initial attachment [29,30]. This causes conformational changes in the capsid structure, causing the minor capsid protein L2 to become exposed and bind to undefined secondary receptors [31,32]. Subsequently, virions are internalized into basal cells to transfer the viral genome to their nucleus through endocytosis, which happens via a mechanism similar to micropinocytosis independent of clatharin, caveolin, lipid rafts, and dynamin [33,34].

The initiation of DNA replication in HPV relies on the early E2 and E1 genes. The HPV viral genome begins to replicate immediately upon entry into epithelial cell [35]. The initial rounds of replication occur upon cell entry with a low copy number (50–100 copies). The viral genome is maintained in these low copy numbers as an episome. The maintenance of low episomal copy number occurs in basal cells, where the low expression of viral protein is caused by E2-mediated suppression of the p97/p105 early promoter, assisting in evading the immune response [36]. E2 inhibits transcription factors’ access to the p97 and p105 promoters and modifies chromatin structure to suppress HPV gene expression [37,38].

### 4.3. E2 Protein-Mediated Assembly of the Replication Initiation Complex

As the cell division cycle progresses, the episomal HPV DNA replicates along with the host cell chromosome. Viral genome DNA replication relies mostly on the host replication machinery. The initial replication that occurs is shown only to utilize E1 and E2 proteins, and studies have indicated that the function of E1 and E2 in promoting DNA replication is conserved across the papillomavirus family [39,40,41]. 

The replication mechanisms in HPV are similar to the replication mechanisms in the *E. coli* system. In *E. coli*, DnaA protein binds to specific binding sites in the origin of DNA replication, oriC and utilizes ATP to form a complex and recruit the DnaB helicase for initiation of DNA replication [42,43]. Similarly in HPV, the E2 protein binds to its binding sites located at the origin of replication. When the replication initiator protein E2 binds to the origin and forms a higher-order complex during the initiation of DNA replication, this complex formation leads to the development of a multi-protein-DNA complex or “Replisome”. By coincidence, the eukaryotic origin recognition complex and the lambda bacteriophage O protein both recognize and activate their respective origins in a similar manner [44,45].

**Figure 6:**
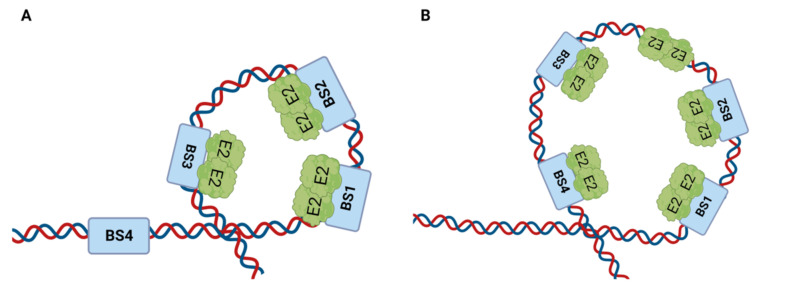
**Loop formation at the origin engineered by the E2 protein**. (**A**) **Small loop formation**. The E2 protein binds to its binding sites at the origin in dimeric form, and this complex formation leads to a loop structure’s formation at the origin of DNA replication. A disk-shaped loop is formed when binding sites 1, 2, and 3 are bound to the E2 protein. (**B**) **Large loop formation**. When E2 is bound to all four binding sites, it attracts more E2 dimers to the complex and larger loop formation occurs.

An electron microscopy analysis of the HPV replication initiation complex found that during HPV DNA replication initiation, two E2 dimers bind to two closely spaced E2 binding sites [45]. The E2–DNA complex instantly transforms into a disk-shaped particle with the addition of the third site in the cluster of three sites, suggesting the presence of a trimer of E2 dimers with DNA looped around the ring (Figure 6A). The inclusion of E2 binding site 4 may lead to the formation of a larger loop, where E2 dimers are bound to all four binding sites (Figure 6B). In either case, additional E2 dimers may join this complex, creating a larger protein–DNA assembly. In order to assist the E1 DNA helicase loading onto the origin and denaturing the origin sequence, the DNA helix may be forced to bend into a tight loop as a result of the loop formation by the E2 dimers. 

It is believed that the E2 protein recruits the helicase E1, possibly by interacting with the E1 via its transactivation domain, which enables activation of the origin of replication. The priming and elongation stages of the replication are carried out by the E1 protein and other cellular replication factors such as DNA polymerases, topoisomerase, and replication protein A (RPA) [18,40,46].

The viral life cycle’s completion occurs in the uppermost epithelial layer after the terminal differentiation of keratinocytes. During this stage, the HPV L1 and L2 genes are expressed and the import of the L1 and L2 capsid proteins triggers virion assembly in the nucleus, which is followed by the release of newly produced virus particles from the epithelial cell surface [47,48,49].

### 4.4. HPV Genome Maintenance and Regulation 

The E1–E2 interaction that mediates replication is sufficient for transient replication in epithelial cells. When cellular division continues, the viral episomes need to be maintained within the daughter cells to avoid degradation [40]. Several viruses use a DNA-binding protein that binds a specific site in the viral DNA and then integrates the genome to host chromosomes. In HPV, the function is performed by the E2 protein [50].

Shortly following the initial replication rounds, the HPV E2 protein uses its transactivation domain to tether the viral DNA to the host chromatin [50,51]. Studies have shown that the E2 protein may need to colocalize with different cellular proteins on the chromosomes for effective maintenance. The most extensively researched anchor for HPV genomes to cellular chromosomes is the human bromodomain protein 4 (Brd4), via E2 [52]. Other than Brd4, Rad50-interacting protein 1 (Rint1), Mitotic kinesin-like protein 2 (MKlp2), DNA topoisomerase 2-binding protein 1 (TopBP1), and cellular DNA helicase ChIR1 have also been shown to interact with the E2 protein as anchors between the viral genome and host cell chromosomes [50,53,54]. 

### 4.5. Transcriptional Regulation

**Figure 7:**
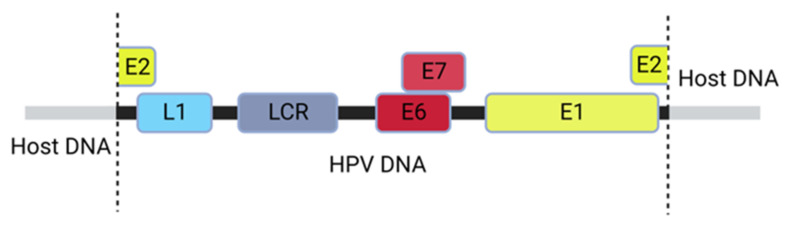
**An illustration of an integrated HPV16 DNA within the host genome**. The integration leads to disruption of the E2 ORF, eliminating the expression of the E2 protein.

**Figure 8:**
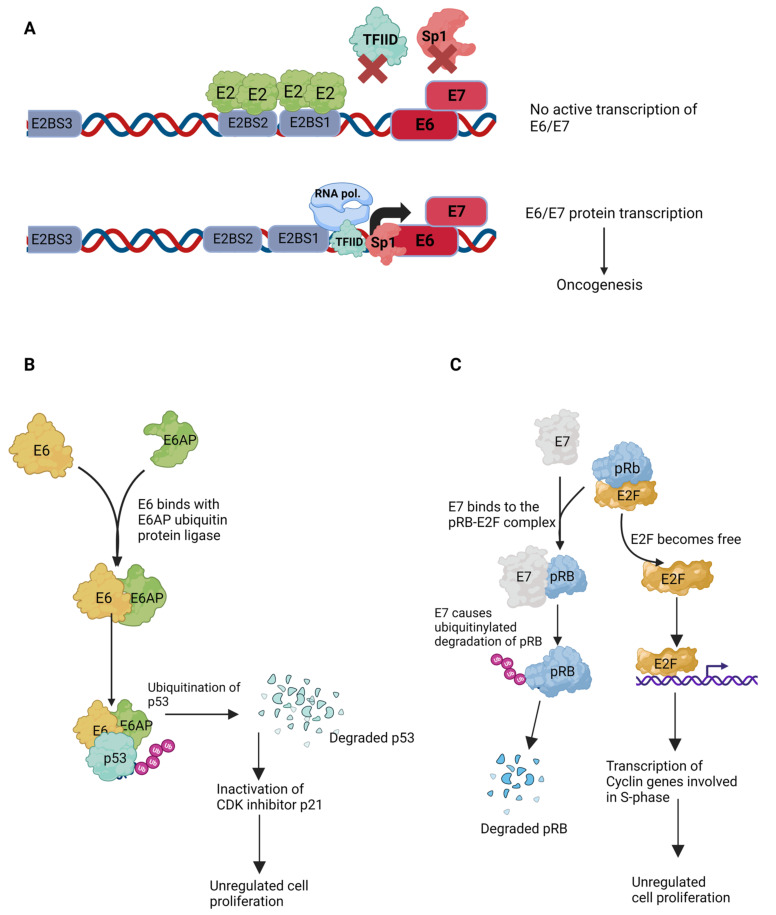
**Pivotal role of E2 protein and the mechanisms of viral oncogenesis.** (**A**). **Transcription repression mechanism of E2**. The promoter p105 of the oncoproteins E6 and E7 lies adjacent to the E2 protein binding sites 1 and 2. When the E2 protein dimer binds at its binding sites, it displaces several transcription factors, such as Sp1 and TFIID, thus preventing transcription of E6/E7. (**B**). **HPV E6 and E7 targeted degradation of p53 and pRb tumor suppressor genes**. E6 protein binds to the cellular ubiquitin-ligase, E6AP, and causes the ubiquitinylated degradation of the p53 protein. This forces the cells through uncontrolled cellular division, evading the preventive checkpoints. (**C**). **HPV E7 protein binds to the pRB-E2F complex**. An essential checkpoint for the cells to travel through the G1-S phase transition. E7 binds to this complex, leading to the ubiquitinylated degradation of the pRb tumor suppressor protein. Degradation of pRb sets the E2F transcription factor free, leading to the unregulated transcription of S-phase cyclin genes and, ultimately, to an unregulated cell cycle.

The replication and transcription mechanisms in HPV are tightly linked because of the E2 protein. HPV E2 functions as a repressor of viral oncogenes. It has been shown to repress E6 and E7 oncogene expression via blockage of the viral promoters p97 and p105 in high-risk HPV types such as 16 and 18 [8,35,55]. This repression is achieved by inhibiting the access of several transcription factors to these promoters. The binding sites for the E2 protein overlap with the early promoter region, and when E2 is bound to its binding sites, BS1 and BS2, the promoter region becomes inaccessible to transcription factors such as Sp1, TFIID, and TFIIB, which ultimately results in silencing of the promoters (Figure 7 and Figure 8A) [56,57,58,59]. The binding site of Sp1, a ubiquitous transcription factor, partially overlaps with one of the E2 binding sites [60,61]. This process is very similar to that of the Lac repressor, which inhibits the transcription of the lac operon genes [62,63]. The E2 protein further influences viral transcription by recruiting and interacting with various cellular host factors. For example, the interaction between E2 and Brd4 could affect E2-mediated transcriptional activation and repression [4]. 

Furthermore, repression or activation of transcription by E2 can depend on the relative positions of E2 binding sites. Studies have shown that interaction with at least two of the binding sites can modulate transcription; however, the configuration of the HPV E2 binding sites is crucial for the successful modulation of transcription. Altering the binding site configuration may lead to altered promoter function [64,65].

### 4.6. HPV Infection Leading to Oncogenesis

Cervical cancer is the fourth most common cancer in women, and ~95% of all cervical cancer cases are HPV-associated [66]. Over the years, the incidence of HPV-associated cervical cancer death has been declining due to cervical cancer screening in the United States and European Union, but not in the developing countries in Asia, Africa, Latin America, and the Caribbean [67]. HPV is the causative agent of several other cancers, such as vulvar, vaginal, anal, penile, and oropharyngeal [68,69]. Most HPV infections may clear spontaneously between 12 and 24 months after the initial infection in the younger population. However, persistent HPV infections, particularly genomic integration, may lead to oncogenesis. It should be noted that the genomic integration of the HPV virus could remain undetectable, and it may cause carcinogenesis years after the original infection. In recent years, studies have found an increasing link between head and neck squamous cell carcinomas and HPV infections [4]. However, how can an HPV infection lead to these various cancers?

During the carcinogenesis of various virus-associated cancers, viral DNA is integrated into the host cell genome [70]. In HPV infection, HPV could integrate into the host genome in a process disrupting the E2 gene sequence as shown in Figure 7. Being the negative regulator of E6/E7 oncogene expression, disruption, truncation, or even silencing of the E2 gene leads to the activation of the oncoproteins [55,71]. HPV-associated cancers are often characterized by the destruction of the p53 and pRb tumor suppressors. In the normal cells, p53 regulates cellular responses to DNA damage and other forms of stress, and pRb is associated with controls of cell division and inhibits unregulated growth [72].

HPV integration sites are also randomly distributed across the human genome. Therefore, further research is required to determine possible patterns for HPV integration in the human genome. In recent years, scientists have begun using Next-Generation Sequencing (NGS) to determine integration signatures [73]. NGS studies found five such signatures. NGS results showed that integration led to truncated forms of the integrated virus, where HPV–chromosome junctions were colinear (2J-COL), nonlinear (2J-NL), multiple hybrid junctions clustering in a single chromosomal region (MJ-CL), scattered over different chromosomal regions (MJ-SC), and episomal (EPI) [73,74,75].

### 4.7. Roles of HPV-Encoded E5, E6, and E7 Proteins

HPV encodes two oncogenes, E6 and E7, which are primarily responsible for inducing carcinogenesis. The E2 protein regulates the expression of both the E6 and E7 genes, as shown in Figure 8A, and actually attenuates or suppresses oncogenesis. The promoters of the overlapping E6 and E7 genes are juxtaposed with the E2BS1 and E2BS2 elements. E2 has high affinity for these two binding sites, as well as E2BS3. Consequently, if sufficient E2 is available, these binding sites remain occupied and hinder the binding of the transcription factors, such as TFIID, from binding to the promoter region. If there is a mutation(s) in BS2 or BS3, E2 binding is inefficient, which allows transcription factors to bind the E6/E7 promoter and induce the transcription of these two oncogenes. In addition, a lack of sufficient E2 protein in the cell, which is the case in the event of genomic integration of the virus and apparent loss of episomal viral DNA, the E2 binding sites remain unoccupied, leading to the expression of the E6 and E7 genes.

The HPV E6 protein is a potent oncogene and functions through the efficient destruction of P53. E6-associated protein (E6AP), which is a ubiquitin protein-ligase also known as UBE3A, has been identified to work with the HPV E6 to degrade p53 by ubiquitination (Figure 8B) [76,77]. The E6 protein binds to a consensus sequence, LxxLL, in the conserved domain of E6AP, creating a heterodimeric complex of E6/E6AP/p53. This complex leads to the ubiquitinylated degradation of the tumor suppressor protein p53. As a result, cells are forced to undergo uncontrolled cellular division and bypass the cell cycle checkpoints. Many in vivo studies demonstrated that interaction with E6AP is essential for developing tumorigenicity in various tumor types [78,79].

Similarly, the HPV E7 protein degrades another tumor suppressor protein, the retinoblastoma protein (pRb). For cells to pass through the G1-S cell cycle boundary, pRb–E2F interaction is a necessary checkpoint. The E2F family of transcription factors remains bound to the pRb protein when cells are not ready to transition to the S-phase of the cell cycle. The E7 protein targets pRb for ubiquitination in HPV-infected cells, releasing the E2F transcription factors, which start transcribing the proteins required in the S-phase (cyclin E, cyclin A, and p16INK4A, an inhibitor of CDK4/6), which causes the cells to enter the S-phase prematurely (Figure 8C) [80].

In addition to causing p53 and pRb degradation, the E6 and E7 proteins can target various host cellular factors and disrupt host signaling pathways. E6 targets other apoptosis-related proteins in addition to p53. These events involve interaction with the host protein Bcl-2 homologous antagonist/killer (BAK), which inhibits intrinsic apoptosis signaling. E6 combines with E6-AP to target BAK for degradation, similar to p53 [80,81,82]. Furthermore, E6 interacts with FADD and caspase-8 to dysregulate the extrinsic apoptosis pathway, which transmits extracellular apoptotic signals from the cell surface [83,84]. It enables the virus to simultaneously block extrinsic signaling from many receptors because these proteins are necessary to enhance signaling from all death recep-tors. Moreover, it has been demonstrated that HPV16 E6 directly binds to the tumor necrosis factor receptor 1, further compromising pro-apoptotic signaling [83,85]. Similar to E6, the E7 oncoprotein is reported to interact with multiple host proteins. The E7 protein has been reported to dysregulate the G1/S-phase checkpoint through several mechanisms, in addition to pRb degradation. E7 binding inhibits the actions of the CDK inhibitors p21CIP1 and p27KIP1, which are reported to play a role in regulating keratinocyte differentiation by causing G1 cell cycle arrest [86,87,88]. Similar to E6, the E7 oncoprotein is reported to interact with multiple host proteins. The E7 protein has been reported to dysregulate the G1/S-phase checkpoint through several mechanisms, in addition to pRb degradation. E7 binding inhibits the actions of the CDK inhibitors p21CIP1 and p27KIP1, which are reported to play a role in regulating keratinocyte differentiation by causing G1 cell cycle arrest [86,87,88].

Furthermore, the HPV E5 protein has also been reported to play a role in oncogenesis. While HPV E5 has relatively limited transforming activity and, consequently, its role throughout the transformation process is not well understood [86,87,88,89], there is solid evidence that it plays a role in the transformation process during infection with bovine papillomaviruses (BPV). E5 regulates cellular signaling in keratinocytes by activating the epidermal growth factor receptor (EGFR)-induced cell proliferation. It stimulates the growth of basal epithelial cells [90,91]. E5 also interferes with keratinocyte growth factor receptor signaling to prevent autophagy and reduce supra-basal keratinocyte proliferation and differentiation [92,93]. All these transformation events lead to uncontrolled proliferation in epithelial cells, which may be an early step in the development of a tumor.

## 5. Summary

HPV is one of the oldest viruses in the world, allowing it to evolve into a large family of viruses. It is generally a silent virus that spreads its infection without major symptoms, potentially leading to various cancers in both men and women.

Despite significant advancements in our understanding of the host pathways that HPV affects, new mechanisms are still being discovered. Therefore, it will be easier to create novel therapeutic drugs if we fully understand the cellular proteins and pathways that HPV disrupts during infection, particularly during carcinogenesis.

The HPV E2 protein plays a significant role in controlling oncogenesis through two mechanisms: (i) regulating viral DNA replication and transcription via binding to its specific binding sites, and (ii) via transcriptionally repressing the E6/E7 oncogene expression, preventing oncogenesis. Therefore, the loss or attenuation of E2 function is pivotal for viral oncogenesis in HPV infection. The loss of expression of the E2 protein occurs due to insertional gene disruption during chromosomal integration of the virus. It can then lead to unregulated expression of the E6 and E7 oncogenes, a preamble to carcinogenesis.

In summary, HPV is a virus that needs further study due to its silent nature and the lack of treatments or a cure. In addition, it is the causative agent of several anogenital cancers and, in recent years, has been linked to other mucosal cancers and head-and-neck cancers. The lack of treatment, combined with the high prevalence in the US and particularly in developing countries with less-than-ideal healthcare systems, calls for a broad-spectrum antiviral drug or vaccine against most high-risk strains of HPV.

## Figures and Tables

**Table 1:** Binding affinities of HPV E2 protein with low-risk and high-risk binding sites as well as binding site sequence variants. Yilmaz et al. [18] analyzed by EMSA and reported the binding affinities of all E2 binding sites of HPV11 and HPV16 and variant binding site sequences of all HPVs reported in Figure 2 with full-length HPV11 E2 (low-risk) or HPV16 E2 (high-risk) ^‡^. Variant nucleotides are indicated in red and lowercase.

HPV Type	Binding Site (BS)	E2 Protein	Binding Site Sequence	Kd (nM)
A. E2 Binding Affinities for HPV11 and HPV16 Binding Sites				
HPV11	BS1	HPV11 E2	ACCG AAAA CGGT	5.8 ± 0.7
HPV11	BS2	HPV11 E2	ACCG AAAA CGGT	5.3 ± 0.4
HPV11	BS3	HPV11 E2	ACCG GTTT CGGT	8.9 ± 0.9
HPV11	BS4	HPV11 E2	ACCG TTTT CGGT	3.9 ± 0.5
HPV16	BS1	HPV16 E2	ACCG AAAC CGGT	7.7 ± 0.7
HPV16	BS2	HPV16 E2	ACCG AAAT CGGT	4.2 ± 0.4
HPV16, 66	BS3	HPV16 E2	ACCG TTTT **g**GGT	≥18
HPV16	BS4	HPV16 E2	ACCG AATT CGGT	3.8 ± 0.4
HPV16	BS3 (reversed)	HPV16 E2	ACCG TTTT **C**GGT	7.0 ± 0.3
HPV16	BS3	HPV11 E2	ACCG TTTT **g**GGT	≥21
**B. E2 Binding Affinities for Variant Binding Sites**				
^†^ HPV18, 30, 33, 39, 45, 51, 53, 59, 68, 70, 97	Various	HPV16 E2	ACCG TTTT **a**GGT	27 ± 2
HPV31	BS2	HPV16 E2	ACCG TTTT **a**GGT	30 ± 3
^†^ HPV34, 35, 56, 58, 73	Various	HPV16 E2	ACCG TTTT **g**G**a**T	67 ± 5
^†^ HPV26, 69, 82	BS3	HPV16 E2	ACCG TTTT **gt**GT	No binding
^†^ HPV16, 66, 67, 82 (BS2)	Various	HPV16 E2	ACCG TTTT **g**GGT	≥18
HPV69 (BS2)	BS2	HPV16 E2	ACCG AAG CGGT	No binding

^†^ Possible evolutionarily linked high-risk types. ^‡^ Taken from the data published by Yilmaz et al. [18].

## Data Availability

Not applicable.

## References

[1] IARC (1995). IARC Monographs on the Evaluation of Carcinogenic Risks to Humans: Human Papillomaviruses. Working Group on the Evaluation of Carcinogenic Risks to Humans.

[2] Graham S.V. (2016). Human Papillomavirus E2 Protein: Linking Replication, Transcription, and RNA Processing. J. Virol..

[3] Muller M., Demeret C. (2012). The HPV E2-Host Protein-Protein Interactions: A Complex Hijacking of the Cellular Network. Open Virol. J..

[4] Kajitani N., Satsuka A., Kawate A., Sakai H. (2012). Productive Lifecycle of Human Papillomaviruses that Depends Upon Squamous Epithelial Differentiation. Front. Microbiol..

[5] Graham S.V. (2010). Human papillomavirus: Gene expression, regulation and prospects for novel diagnostic methods and antiviral therapies. Future Microbiol..

[6] Graham Sheila V. (2017). The human papillomavirus replication cycle, and its links to cancer progression: A comprehensive review. Clin. Sci..

[7] D’Abramo C.M., Archambault J. (2011). Small molecule inhibitors of human papillomavirus protein—Protein interactions. Open Virol. J..

[8] Ribeiro A.L., Caodaglio A.S., Sichero L. (2018). Regulation of HPV transcription. Clinics.

[9] Ong C.K., Chan S.Y., Campo M.S., Fujinaga K., Mavromara-Nazos P., Labropoulou V., Pfister H., Tay S.K., ter Meulen J., Villa L.L. (1993). Evolution of human papillomavirus type 18: An ancient phylogenetic root in Africa and intratype diversity reflect coevolution with human ethnic groups. J. Virol..

[10] Zur Hausen H., Gissmann L., Steiner W., Dippold W., Dreger I. (1976). Human papilloma viruses and cancer. Comparative Leukemia Research 1975.

[11] Bzhalava D., Eklund C., Dillner J. (2015). International standardization and classification of human papillomavirus types. Virology.

[12] De Koning M.N., Quint K.D., Bruggink S.C., Gussekloo J., Bouwes Bavinck J.N., Feltkamp M.C., Quint W.G., Eekhof J.A. (2015). High prevalence of cutaneous warts in elementary school children and the ubiquitous presence of wart-associated human papillomavirus on clinically normal skin. Br. J. Dermatol..

[13] Egawa N., Egawa K., Griffin H., Doorbar J. (2015). Human Papillomaviruses; Epithelial Tropisms, and the Development of Neoplasia. Viruses.

[14] Agalliu I., Gapstur S., Chen Z., Wang T., Anderson R.L., Teras L., Kreimer A.R., Hayes R.B., Freedman N.D., Burk R.D. (2016). Associations of Oral α-, β-, and γ-Human Papillomavirus Types With Risk of Incident Head and Neck Cancer. JAMA Oncol..

[15] Sias C., Salichos L., Lapa D., Del Nonno F., Baiocchini A., Capobianchi M.R., Garbuglia A.R. (2019). Alpha, Beta, gamma human PapillomaViruses (HPV) detection with a different sets of primers in oropharyngeal swabs, anal and cervical samples. Virol. J..

[16] Braaten K.P., Laufer M.R. (2008). Human Papillomavirus (HPV), HPV-Related Disease, and the HPV Vaccine. Rev. Obstet. Gynecol..

[17] Burd E.M. (2003). Human Papillomavirus and Cervical Cancer. Clin. Microbiol. Rev..

[18] Yilmaz G., Biswas-Fiss E.E., Biswas S.B. (2018). Genetic variations in the DNA replication origins of human papillomavirus family correlate with their oncogenic potential. Biochim. Biophys. Acta Gen. Subj..

[19] Sakai H., Yasugi T., Benson J.D., Dowhanick J.J., Howley P.M. (1996). Targeted mutagenesis of the human papillomavirus type 16 E2 transactivation domain reveals separable transcriptional activation and DNA replication functions. J. Virol..

[20] Antson A.A., Burns J.E., Moroz O.V., Scott D.J., Sanders C.M., Bronstein I.B., Dodson G.G., Wilson K.S., Maitland N.J. (2000). Structure of the intact transactivation domain of the human papillomavirus E2 protein. Nature.

[21] Hegde R.S. (2002). The Papillomavirus E2 Proteins: Structure, Function, and Biology. Annu. Rev. Biophys. Biomol. Struct..

[22] McBride A.A. (2013). The papillomavirus E2 proteins. Virology.

[23] Lace M.J., Anson J.R., Thomas G.S., Turek L.P., Haugen T.H. (2008). The E8^E2 gene product of human papillomavirus type 16 represses early transcription and replication but is dispensable for viral plasmid persistence in keratinocytes. J. Virol..

[24] Hawley-Nelson P., Androphy E.J., Lowy D.R., Schiller J.T. (1988). The specific DNA recognition sequence of the bovine papillomavirus E2 protein is an E2-dependent enhancer. EMBO J.

[25] Bedrosian C.L., Bastia D. (1990). The DNA-binding domain of HPV-16 E2 protein interaction with the viral enhancer: Protein-induced DNA bending and role of the nonconserved core sequence in binding site affinity. Virology.

[26] Thain A., Webster K., Emery D., Clarke A.R., Gaston K. (1997). DNA Binding and Bending by the Human Papillomavirus Type 16 E2 Protein. J. Biol. Chem..

[27] Yilmaz G., Biswas-Fiss E.E., Biswas S.B. (2023). Sequence-Dependent Interaction of the Human Papillomavirus E2 Protein with the DNA Elements on Its DNA Replication Origin. Int. J. Mol. Sci..

[28] Aksoy P., Gottschalk E.Y., Meneses P.I. (2017). HPV entry into cells. Mutat. Res./Rev. Mutat. Res..

[29] Giroglou T., Florin L., Schäfer F., Streeck R.E., Sapp M. (2001). Human Papillomavirus Infection Requires Cell Surface Heparan Sulfate. J. Virol..

[30] Letian T., Tianyu Z. (2010). Cellular receptor binding and entry of human papillomavirus. Virol. J..

[31] Wang J.W., Roden R.B.S. (2013). L2, the minor capsid protein of papillomavirus. Virology.

[32] Schelhaas M., Shah B., Holzer M., Blattmann P., Kühling L., Day P.M., Schiller J.T., Helenius A. (2012). Entry of Human Papillomavirus Type 16 by Actin-Dependent, Clathrin- and Lipid Raft-Independent Endocytosis. PLoS Pathog..

[33] Spoden G., Freitag K., Husmann M., Boller K., Sapp M., Lambert C., Florin L. (2008). Clathrin- and Caveolin-Independent Entry of Human Papillomavirus Type 16—Involvement of Tetraspanin-Enriched Microdomains (TEMs). PLoS ONE.

[34] Kuo S.R., Liu J.S., Broker T.R., Chow L.T. (1994). Cell-free replication of the human papillomavirus DNA with homologous viral E1 and E2 proteins and human cell extracts. J. Biol. Chem..

[35] Romanczuk H., Thierry F., Howley P.M. (1990). Mutational analysis of cis elements involved in E2 modulation of human papillomavirus type 16 P97 and type 18 P105 promoters. J. Virol..

[36] Smith J.A., Haberstroh F.S., White E.A., Livingston D.M., DeCaprio J.A., Howley P.M. (2014). SMCX and components of the TIP60 complex contribute to E2 regulation of the HPV E6/E7 promoter. Virology.

[37] Smith J.A., White E.A., Sowa M.E., Powell M.L.C., Ottinger M., Harper J.W., Howley P.M. (2010). Genome-wide siRNA screen identifies SMCX, EP400, and Brd4 as E2-dependent regulators of human papillomavirus oncogene expression. Proc. Natl. Acad. Sci. USA.

[38] Chiang C.M., Ustav M., Stenlund A., Ho T.F., Broker T.R., Chow L.T. (1992). Viral E1 and E2 proteins support replication of homologous and heterologous papillomaviral origins. Proc. Natl. Acad. Sci. USA.

[39] McBride A.A. (2008). Replication and partitioning of papillomavirus genomes. Adv. Virus Res..

[40] Vecchio A.M.D., Romanczuk H., Howley P.M., Baker C.C. (1992). Transient replication of human papillomavirus DNAs. J. Virol..

[41] Bramhill D., Kornberg A. (1988). A model for initiation at origins of DNA replication. Cell.

[42] Funnell B.E., Baker T.A., Kornberg A. (1987). In vitro assembly of a prepriming complex at the origin of the Escherichia coli chromosome. J. Biol. Chem..

[43] Alfano C., McMacken R. (1989). Ordered Assembly of Nucleoprotein Structures at the Bacteriophage λ Replication Origin during the Initiation of DNA Replication. J. Biol. Chem..

[44] Gaczynska M., Osmulski P.A., Jiang Y., Lee J.-K., Bermudez V., Hurwitz J. (2004). Atomic force microscopic analysis of the binding of the Schizosaccharomyces pombe origin recognition complex and the spOrc4 protein with origin DNA. Proc. Natl. Acad. Sci. USA.

[45] Sim J., Ozgur S., Lin B.Y., Yu J.-H., Broker T.R., Chow L.T., Griffith J. (2008). Remodeling of the Human Papillomavirus Type 11 Replication Origin into Discrete Nucleoprotein Particles and Looped Structures by the E2 Protein. J. Mol. Biol..

[46] Berg M., Stenlund A. (1997). Functional interactions between papillomavirus E1 and E2 proteins. J. Virol..

[47] Becker K.A., Florin L., Sapp C., Sapp M. (2003). Dissection of human papillomavirus type 33 L2 domains involved in nuclear domains (ND) 10 homing and reorganization. Virology.

[48] Darshan M.S., Lucchi J., Harding E., Moroianu J. (2004). The L2 Minor Capsid Protein of Human Papillomavirus Type 16 Interacts with a Network of Nuclear Import Receptors. J. Virol..

[49] Florin L., Sapp C., Streeck R.E., Sapp M. (2002). Assembly and Translocation of Papillomavirus Capsid Proteins. J. Virol..

[50] You J., Croyle J.L., Nishimura A., Ozato K., Howley P.M. (2004). Interaction of the bovine papillomavirus E2 protein with Brd4 tethers the viral DNA to host mitotic chromosomes. Cell.

[51] McBride A.A., Oliveira J.G., McPhillips M.G. (2006). Partitioning viral genomes in mitosis: Same idea, different targets. Cell Cycle.

[52] Iftner T., Haedicke-Jarboui J., Wu S.-Y., Chiang C.-M. (2017). Involvement of Brd4 in different steps of the papillomavirus life cycle. Virus Res..

[53] Donaldson M.M., Boner W., Morgan I.M. (2007). TopBP1 Regulates Human Papillomavirus Type 16 E2 Interaction with Chromatin. J. Virol..

[54] Parish J.L., Bean A.M., Park R.B., Androphy E.J. (2006). ChlR1 Is Required for Loading Papillomavirus E2 onto Mitotic Chromosomes and Viral Genome Maintenance. Mol. Cell.

[55] McBride A.A., Warburton A. (2017). The role of integration in oncogenic progression of HPV-associated cancers. PLoS Pathog..

[56] Bernard B.A., Bailly C., Lenoir M.C., Darmon M., Thierry F., Yaniv M. (1989). The human papillomavirus type 18 (HPV18) E2 gene product is a repressor of the HPV18 regulatory region in human keratinocytes. J. Virol..

[57] Dong G., Broker T.R., Chow L.T. (1994). Human papillomavirus type 11 E2 proteins repress the homologous E6 promoter by interfering with the binding of host transcription factors to adjacent elements. J. Virol..

[58] Nishimura A., Ono T., Ishimoto A., Dowhanick J.J., Frizzell M.A., Howley P.M., Sakai H. (2000). Mechanisms of human papillomavirus E2-mediated repression of viral oncogene expression and cervical cancer cell growth inhibition. J. Virol..

[59] Rank N.M., Lambert P.F. (1995). Bovine papillomavirus type 1 E2 transcriptional regulators directly bind two cellular transcription factors, TFIID and TFIIB. J. Virol..

[60] Jang M.K., Kwon D., McBride A.A. (2009). Papillomavirus E2 proteins and the host BRD4 protein associate with transcriptionally active cellular chromatin. J. Virol..

[61] Võsa L., Sudakov A., Remm M., Ustav M., Kurg R. (2012). Identification and analysis of papillomavirus E2 protein binding sites in the human genome. J. Virol..

[62] Gilbert W. (1972). The lac repressor and the lac operator. Ciba Found. Symp..

[63] Lin S.Y., Riggs A.D. (1970). Lac repressor binding to DNA not containing the lac operator and to synthetic poly dAT. Nature.

[64] Kovelman R., Bilter G.K., Glezer E., Tsou A.Y., Barbosa M.S. (1996). Enhanced transcriptional activation by E2 proteins from the oncogenic human papillomaviruses. J. Virol..

[65] Tan S.-H., Gloss B., Bernard H.-U. (1992). During negative regulation of the human papillomavirus-16E6 promoter, the viral E2 protein can displace Sp1 from a proximal promoter element. Nucleic Acids Res..

[66] Van Dyne E.A., Henley S.J., Saraiya M., Thomas C.C., Markowitz L.E., Benard V.B. (2018). Trends in Human Papillomavirus-Associated Cancers—United States, 1999–2015. MMWR Morb. Mortal. Wkly. Rep..

[67] Vaccarella S., Laversanne M., Ferlay J., Bray F. (2017). Cervical cancer in Africa, Latin America and the Caribbean and Asia: Regional inequalities and changing trends. Int. J. Cancer.

[68] Okunade K.S. (2020). Human papillomavirus and cervical cancer. J. Obstet. Gynaecol..

[69] Oyervides-Muñoz M.A., Pérez-Maya A.A., Rodríguez-Gutiérrez H.F., Gómez-Macias G.S., Fajardo-Ramírez O.R., Treviño V., Barrera-Saldaña H.A., Garza-Rodríguez M.L. (2018). Understanding the HPV integration and its progression to cervical cancer. Infect. Genet. Evol..

[70] Williams V.M., Filippova M., Soto U., Duerksen-Hughes P.J. (2011). HPV-DNA integration and carcinogenesis: Putative roles for inflammation and oxidative stress. Future Virol..

[71] Zhou L., Qiu Q., Zhou Q., Li J., Yu M., Li K., Xu L., Ke X., Xu H., Lu B. (2022). Long-read sequencing unveils high-resolution HPV integration and its oncogenic progression in cervical cancer. Nat. Commun..

[72] Yim E.K., Park J.S. (2005). The role of HPV E6 and E7 oncoproteins in HPV-associated cervical carcinogenesis. Cancer Res. Treat..

[73] Holmes A., Lameiras S., Jeannot E., Marie Y., Castera L., Sastre-Garau X., Nicolas A. (2016). Mechanistic signatures of HPV insertions in cervical carcinomas. NPJ Genom. Med..

[74] Kamal M., Lameiras S., Deloger M., Morel A., Vacher S., Lecerf C., Dupain C., Jeannot E., Girard E., Baulande S. (2021). Human papilloma virus (HPV) integration signature in Cervical Cancer: Identification of MACROD2 gene as HPV hot spot integration site. Br. J. Cancer.

[75] Mainguené J., Vacher S., Kamal M., Hamza A., Masliah-Planchon J., Baulande S., Ibadioune S., Borcoman E., Cacheux W., Calugaru V. (2022). Human papilloma virus integration sites and genomic signatures in head and neck squamous cell carcinoma. Mol. Oncol..

[76] Scheffner M., Huibregtse J.M., Vierstra R.D., Howley P.M. (1993). The HPV-16 E6 and E6-AP complex functions as a ubiquitin-protein ligase in the ubiquitination of p53. Cell.

[77] Scheffner M., Werness B.A., Huibregtse J.M., Levine A.J., Howley P.M. (1990). The E6 oncoprotein encoded by human papillomavirus types 16 and 18 promotes the degradation of p53. Cell.

[78] Kelley M.L., Keiger K.E., Lee C.J., Huibregtse J.M. (2005). The global transcriptional effects of the human papillomavirus E6 protein in cervical carcinoma cell lines are mediated by the E6AP ubiquitin ligase. J. Virol..

[79] Nguyen M., Song S., Liem A., Androphy E., Liu Y., Lambert P.F. (2002). A mutant of human papillomavirus type 16 E6 deficient in binding alpha-helix partners displays reduced oncogenic potential in vivo. J. Virol..

[80] Boyer S.N., Wazer D.E., Band V. (1996). E7 protein of human papilloma virus-16 induces degradation of retinoblastoma protein through the ubiquitin-proteasome pathway. Cancer Res..

[81] Thomas M., Banks L. (1998). Inhibition of Bak-induced apoptosis by HPV-18 E6. Oncogene.

[82] Thomas M., Banks L. (1999). Human papillomavirus (HPV) E6 interactions with Bak are conserved amongst E6 proteins from high and low risk HPV types. J. Gen. Virol..

[83] Filippova M., Parkhurst L., Duerksen-Hughes P.J. (2004). The Human Papillomavirus 16 E6 Protein Binds to Fas-associated Death Domain and Protects Cells from Fas-triggered Apoptosis. J. Biol. Chem..

[84] Tungteakkhun S.S., Filippova M., Neidigh J.W., Fodor N., Duerksen-Hughes P.J. (2008). The interaction between human papillomavirus type 16 and FADD is mediated by a novel E6 binding domain. J. Virol..

[85] Filippova M., Song H., Connolly J.L., Dermody T.S., Duerksen-Hughes P.J. (2002). The Human Papillomavirus 16 E6 Protein Binds to Tumor Necrosis Factor (TNF) R1 and Protects Cells from TNF-induced Apoptosis. J. Biol. Chem..

[86] Funk J.O., Waga S., Harry J.B., Espling E., Stillman B., Galloway D.A. (1997). Inhibition of CDK activity and PCNA-dependent DNA replication by p21 is blocked by interaction with the HPV-16 E7 oncoprotein. Genes Dev..

[87] Jones D.L., Alani R.M., Münger K. (1997). The human papillomavirus E7 oncoprotein can uncouple cellular differentiation and proliferation in human keratinocytes by abrogating p21<sup>Cip1</sup>-mediated inhibition of cdk2. Genes Dev..

[88] Zerfass-Thome K., Zwerschke W., Mannhardt B., Tindle R., Botz J.W., Jansen-Dürr P. (1996). Inactivation of the cdk inhibitor p27KIP1 by the human papillomavirus type 16 E7 oncoprotein. Oncogene.

[89] DiMaio D., Petti L.M. (2013). The E5 proteins. Virology.

[90] Pim D., Collins M., Banks L. (1992). Human papillomavirus type 16 E5 gene stimulates the transforming activity of the epidermal growth factor receptor. Oncogene.

[91] Straight S.W., Hinkle P.M., Jewers R.J., McCance D.J. (1993). The E5 oncoprotein of human papillomavirus type 16 transforms fibroblasts and effects the downregulation of the epidermal growth factor receptor in keratinocytes. J. Virol..

[92] Barbaresi S., Cortese M.S., Quinn J., Ashrafi G.H., Graham S.V., Campo M.S. (2010). Effects of human papillomavirus type 16 E5 deletion mutants on epithelial morphology: Functional characterization of each transmembrane domain. J. Gen. Virol..

[93] Belleudi F., Leone L., Purpura V., Cannella F., Scrofani C., Torrisi M.R. (2011). HPV16 E5 affects the KGFR/FGFR2b-mediated epithelial growth through alteration of the receptor expression, signaling and endocytic traffic. Oncogene.

